# Spontaneous Mnemonic Strategy Use Under Proactive Interference Is Related to Better Recall But Does Not Protect from Accumulating Interference

**DOI:** 10.1177/17470218261454265

**Published:** 2026-05-20

**Authors:** Matti Laine, Tilda Eräste, Anton Kunnari

**Affiliations:** 1 Department of Psychology, Åbo Akademi University, Finland; 2 Unit of Psychology, University of Oulu, Turku, Finland; 3 Department of Psychology and Logopedics, University of Helsinki, Finland

**Keywords:** memory strategy, mnemonics, proactive interference, word list recall

## Abstract

Proactive interference (PI) is a key mechanism in forgetting, making it important to examine factors that may alleviate its detrimental effects. In a preregistered online study with 207 English-speaking 18- to 60-year-old adults, we probed block-by-block spontaneous mnemonic strategy use in a categorised word list recall task with increasing PI, followed by release from PI. Based on the blockwise self-reports, participants were classified as no strategy users, maintenance strategy users (e.g., repetition), or manipulation strategy users (e.g., grouping, creating a story). Strategy users formed a majority and showed better recall than no strategy users, with the highest recall rates in manipulation strategy users. The detrimental effects of PI on word recall were similar in the three subgroups. Self-reports suggested increased shifts of the selected strategy when moving from the last PI block to release from PI. Altogether, the present results indicate that self-generated memory strategies are (a) common in adults, (b) flexibly used when faced with a qualitative change in task demands, (c) associated with better memory performance especially concerning more sophisticated strategies involving mental manipulation of the memoranda, but (d) do not prevent the buildup of PI as they may not provide enough differentiation between current and previous category members.

## Introduction

Proactive interference (PI) is a well-known phenomenon where materials learned earlier impair memory for subsequent, related materials. An everyday life example would be a difficulty in remembering a new password as it gets mixed up with the old ones. PI is an important aspect of memory function that has long been considered a main contributing factor in forgetting (Miller, 2021; Underwood, 1957). PI effects are modulated by different factors. One of these factors is mnemonic strategy use: previous research has found that employment of externally instructed mnemonic strategies under PI leads to improved recall (for more recent studies, see Bass & Oswald, 2014; Massen & Vaterrodt-Plünnecke, 2006). However, less is known about people’s spontaneous memory strategies under PI, their association with memory performance and the evolution of strategy use during the task. The present preregistered experiment examined these issues by studying memory for categorised word lists (the Brown-Peterson paradigm), one of the common techniques employed in PI research, and by collecting open strategy reports after retrieval of each word list.

Accumulating PI with to-be-remembered semantically related word lists, for example animal names, is manifested as decreased recall and intrusions from the previously studied and recalled lists, and also as longer recall latencies (for a review, see Kliegl & Bäuml, 2021). However, when a change of semantic category takes place for example from lists of animal names to occupations, release from PI is observed as a rebound of recall performance (Wickens, 1970).

The cognitive mechanisms underlying the buildup of PI have been widely discussed, and they have been related to both encoding and retrieval. As regards encoding, waning attention has been postulated based on decreasing pupil size during encoding (Engle, 1975; Johansson et al., 2018), albeit this may be related to increasing task familiarity rather than accumulating interference. Moreover, Kane and Engle (2000) found that performing a secondary task during encoding led to increased PI in participants with high working memory capacity (those with low capacity were assumed to have exhausted their attentional capabilities even without the secondary task). This suggests that under PI, attentional resources are engaged during encoding by high working memory capacity individuals. These resources may be used to actively inhibit items from previous related word lists or to generate a “time tag” to differentiate the lists from one another (Kane & Engle, 2000). Regarding retrieval, problems in discriminating between the current items and earlier related stimuli have been hypothesised because recall latencies get longer along the task, suggesting that participants go through a wider, more diffuse search set when trying to recall the items of the latest list (Wixted & Rohrer, 1993). This is in line with the cue-overload theory, where memory performance declines as more and more stimuli share the same retrieval cues (Watkins & Watkins, 1976). The PI study by Kane and Engle (2000) cited above found that dual tasking increased PI in high working memory capacity participants, also at retrieval, speaking for the role of attentional processes at this stage. Release from PI has also been attributed to retrieval, where the new category provides a distinctive retrieval cue that enables the exclusion of the previous lists. The shorter recall latencies observed by Wixted and Rohrer (1993) after release from PI suggest that the search set during retrieval becomes smaller. In line with a retrieval account, Engle (1975) observed that release from PI was not accompanied by increased pupil size during encoding.

How could the use of spontaneous (non-prompted) mnemonic strategies affect PI? In general, spontaneous memory strategies, particularly those involving manipulation of the memoranda such as grouping stimuli or creating associations, have been associated with superior recall in different memory domains, including episodic memory (Dunlosky & Hertzog, 2001; Dunlosky & Kane, 2007; Unsworth et al., 2019; Waris et al., 2021a), working memory (Brown & Wesley, 2013; Forsberg et al., 2020; Laine et al., 2018; McNamara & Scott, 2001; Waris et al., 2021b) and prospective memory (Laine et al., 2024b; Reese-Melancon et al., 2019). Also, under PI, spontaneous mnemonic strategies could help in keeping focus on the current stimulus list and providing more distinctive cues for retrieval even within a semantic category. One may also speculate whether the buildup of competition between recent and previous memories would prompt strategy use to counteract the interference participants are experiencing. Some hints on the positive effects of self-generated strategies even in the face of PI are provided by the study of Bass and Oswald (2014), who reported pervasive spontaneous strategy use with word list recall under PI in their control group. Interestingly, the decrease in word recall because of PI was smallest in those control participants who, at the end of the task, reported visualising or creating a story of the to-be-remembered items. However, the subgroups of different spontaneous strategy users were too small to enable any statistical analyses. Regarding their experimental group that was instructed to use a sophisticated mnemonic technique, the method of loci, Bass and Oswald (2014) found significantly less PI in terms of word recall than in their non-instructed control group. All in all, the role of spontaneous strategy use under accumulating PI and the possible within-task evolution of strategy use is not known.

We preregistered two alternative hypotheses on the strategy-related advantage in PI: (1) Spontaneous (non-prompted) strategy use as compared with no strategy use is related to better memory performance, but the relative performance decrement through the buildup of PI is similar. In other words, the PI-related gradual decrease in word recall as a function of task block follows a parallel course in strategy users versus non-users. This would also be the case for possible intrusion errors. This pattern would echo the more or less constant recall advantage of strategy users versus non-users over task blocks, for example, in the word list learning study by Laine et al. (2024a). (2) Strategy users are less sensitive to accumulating PI, so the decreasing word recall curves diverge between strategy users and non-users over the PI blocks. Thus, strategy non-users exhibit a more marked performance decrease over the PI blocks than strategy users do. This should also be true for possible intrusion errors, which would show a relatively larger increase under PI in strategy non-users than in strategy users.

In sum, our first hypothesis predicts a stable strategy-related advantage in recall that is independent of the accumulation of PI. The second hypothesis presupposes that spontaneous strategy use attenuates the negative effects of gradually increasing PI on recall.

## Materials and Methods

### Participants

The anonymous participants of this online study were recruited via the crowdsourcing site Prolific (https://www.prolific.co/). The following filters were applied on Prolific: living in the United Kingdom or Ireland, aged between 18 and 60 years, first language English, have not participated in the pilot study for this experiment, alcohol consumption of 13 or fewer units per week, no current diagnosis of a mental health condition (“Are you currently diagnosed with a mental health condition?”), no medical diagnosis of dyslexia (“Have you received a medical diagnosis for dyslexia?”), no clinical diagnosis of autism spectrum disorder (“Have you received a formal clinical diagnosis of autism spectrum disorder, made by a psychiatrist, psychologist, or other qualified medical specialist? This includes Asperger’s syndrome, Autism Disorder, High Functioning Autism or Pervasive Developmental Disorder”), and no suspected or diagnosed ADHD/ADD by self-report (“Do you consider yourself to have attention deficit disorder (ADD)/attention deficit hyperactivity disorder (ADHD)?”).

The preregistered goal was to collect complete data of 240 participants (60 per counterbalanced stimulus set), which was fulfilled. This goal was set on the basis of our recent strategy-related memory studies, where similar sample sizes had secured enough observations for comparisons between main spontaneous strategy types in task blocks (Laine et al., 2024a, 2024b). For the collected sample of 240 participants, further exclusions were made on the basis of our own questions: participant reports use of external aids (e.g., making notes during the task to aid memory), reports ailments that can affect their current mental abilities (neurologic, neuropsychiatric, psychiatric, use of medication, alcohol, or drugs), or fails on 5 or more arithmetic distractor tasks presented after each word list. Following these criteria, a total of 33 participants were left out from the final analyses, including 16 based on our own questions (use of external aids *n* = 4; health-related reason *n* = 6; problems encountered with the recall tasks *n* = 5; data missing on relevant background variables *n* = 1), 6 because of technical problems that affected their data, 9 univariate outliers (scoring 3 times the interquartile range above or below the first or the third quartile in a given dependent variable), and 2 because of missing strategy-related data. Thus, the final sample consisted of 207 participants. Demographic information of the final sample is given in Table 1.

**Table 1.** Background Characteristics of the Sample (*n* = 207).

Gender (F/M/other)	136/70/1
Age, *M* (*SD*)	40.25 (10.38)
Education level (%)	Primary school: 1 (0.5%) Secondary school up to 16 years: 23 (11.1%) Higher or secondary or further education (A-levels, BTEC, etc.): 46 (22.2%) College or university: 98 (47.3%) Post-graduate degree: 39 (18.8%)

*Note. SD* = standard deviation. BTEC = Business and Technology Education Council.

### Study Design and Procedure

The present study employed the Brown-Peterson paradigm, where participants studied multiple word lists. Each list was followed by a distractor task, a recall phase, an open strategy report, and a subjective difficulty rating, together forming a task block. The first three word lists aimed at creating PI were semantically related (animal names), while the fourth list with release from PI represented a different semantic category (names of countries or occupations). The experimenter-controlled independent variable was the task block (blocks 1–3: PI buildup; block 4: release from PI), whereas the natural independent variables per task block were the main spontaneous strategy type (no strategy, maintenance strategy, manipulation strategy), level of detail in strategy report, and the number of primary strategies employed. The main dependent variable was the correctness of recall for each item in each task block (see below for the coding of correctness).

The study began with a short list of background questions concerning the participants’ current health status (neurological, psychiatric, or neurodevelopmental disorders and alcohol, drug, or medication use that may affect their current mental abilities), level of education, and self-evaluated memory function.

After filling out the questionnaire, the PI task began. Figure 1 illustrates the structure of a task block. In this task, the participants were instructed to memorise a list of 10 words. The words were shown on screen one by one in randomised order for 2 s with a 2-s inter-stimulus interval. After the list had been shown, the participants were asked to solve three simple arithmetic operations to prevent the words from being processed in their working memory (for the time taken to solve these, see the Supplemental Table S2). After solving this distractor task at their own pace, the participants were instructed to type in as many words from the previously presented word list as they could remember in a set of empty response boxes that appeared on screen one by one. The participant could not see previously entered words. It was emphasised in the instructions that the order of the recalled words was not important. After the recall phase, a strategy report was given (“Please report in as much detail as possible if you used some strategy to help you in learning the word list. That is, how did you try to memorise the words?”). After finishing the strategy report, the participants were asked to rate how difficult it was to remember the words from the previous list on a 7-point Likert scale (1 = *very easy*, 7 = *very difficult*). This procedure was repeated four times with a different word list on each round. The first 3 lists consisted of animal names and the fourth word list included either 10 countries or 10 occupations, depending on which day the participant had participated in the study. The selection procedure and the word lists are described below.

**Figure 1.**
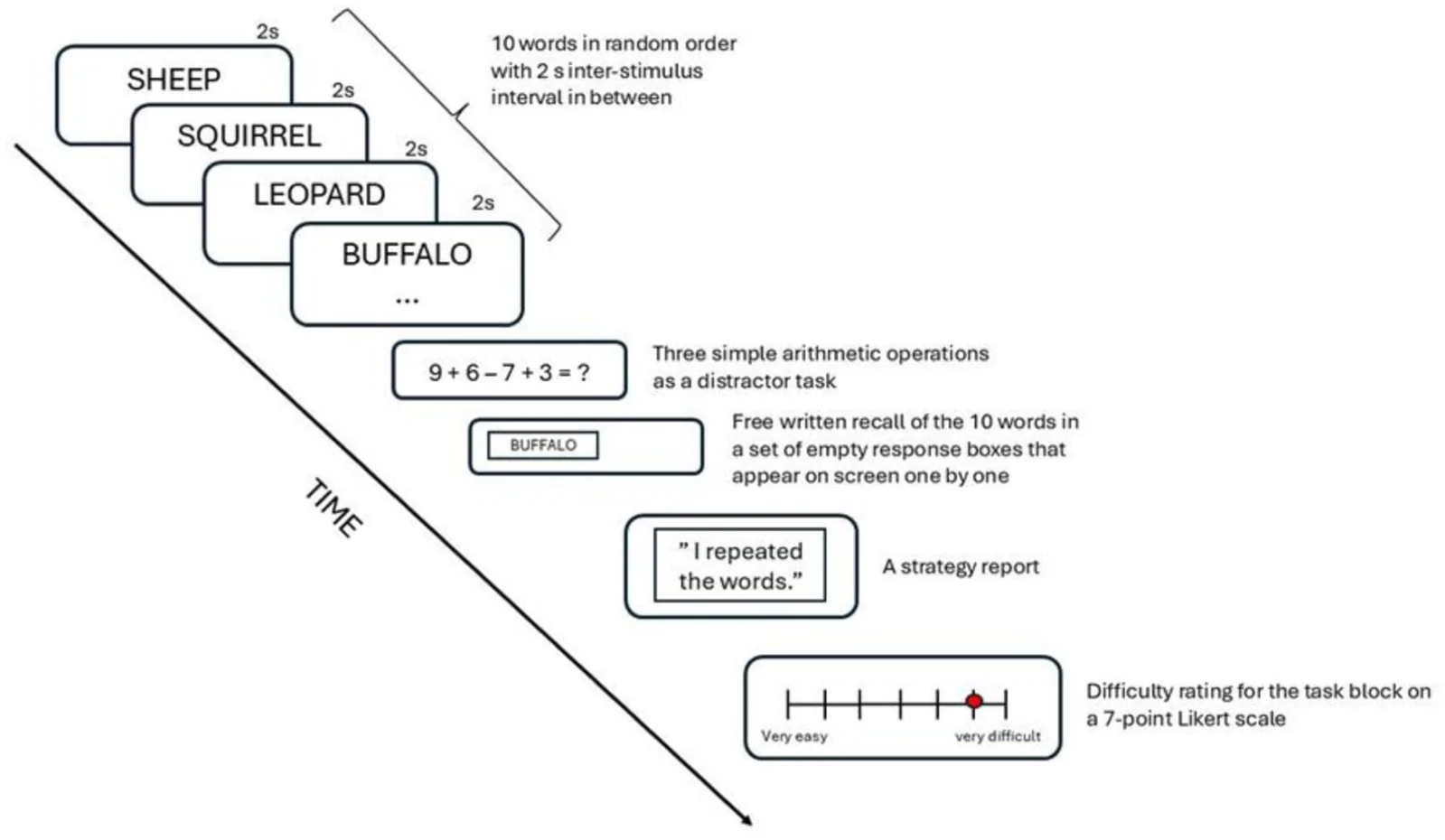
The structure of a task block in the PI task. This procedure was repeated four times with a different word list on each round. The first 3 lists consisted of animal names (the PI task blocks), and the fourth word list included either 10 countries or 10 occupations (the task block with release from PI). *Note.* PI = proactive interference.

The experiment was programmed using PsychoPy Builder version 2023.2.1 (Peirce et al., 2019) and run online via Pavlovia (https://pavlovia.org/). The data were collected in September 2023 in four separate parts from Monday to Thursday. Every day at 10 am (UTC), 60 slots were opened for the study on a first-come, first-served basis. The only difference between these four parts was the set of word lists used (see the selection process of word list sets in the “Test Materials” section). Each participant was able to attend the study only on 1 of these 4 days. The entire session lasted approximately 15 min, and the participants were reimbursed with £2 upon completion.

### Test Materials

#### Word List Sets

The word lists were taken from a study by Kane and Engle (2000). The original sets were compiled as follows: (a) 40 words were chosen from 3 distinct taxonomic groups in the Battig and Montague (1969) category norms, which included animals, occupations, and countries, (b) the chosen words consisted of single words with 10 or fewer letters and were ranked below the top 12 strongest associates within their respective category, (c) in each category, the 40 words were divided into 4 sets of 10 words each, striving to closely match the associative strengths within these subsets, (d) from these subsets, a total of 12 full stimulus sets were created, each consisting of 40 words.

In the current study, 4 of these 12 original stimulus sets were used (see Appendix A in Kane & Engle, 2000). Two words from the original lists were changed (“donkey” → “horse” due to its close similarity to another target word, “monkey”; “Bolivia” → “Norway” as it, for some reason, appeared twice on the original list). Each set consisted of three subsets from the animal category (PI buildup lists) and one subset from either the occupation or the country category (a PI release list). Thus, two of the animal PI buildup lists were followed by the occupation PI release list, and the other two by the country PI release list. This systematic variation ensured that the results did not reflect possible idiosyncrasies of a specific stimulus set. The participants were distributed among these four sets in the following way: set A *n* = 46; set B *n* = 53; set C *n* = 58; set D *n* = 50.

The trial-level data used in the analyses were created with an algorithm that checked whether each presented word (trial) was present in the list of responses participants gave after each block, with the ability to detect correctly recalled words with minor typing errors. Each response word and the target word were cross-checked for each consecutive letter pair so that the percentage of pairs of the target *present* in the response quantified correctness, and the percentage of pairs of the response *absent* in the target quantified incorrectness. This double-layered checking prevented very similar words from getting scored incorrectly. For example, a target word “ox” is completely present (100% correct) in a response “fox,” but the response also contains an additional letter pair (“fo,” “ox”), flagging the response as 50% incorrect. Perfect responses (100% correct, 0% incorrect) were automatically coded as correct, and responses with a high amount of correctness and a low amount of incorrectness were inspected and scored manually. See the OSF materials for the code.

#### Memory Question

We examined the participants’ subjective evaluation of their memory by asking them a single question: “How would you rate your memory in terms of the kinds of memory problems that you may have?” (see the Memory Functioning Questionnaire by Gilewski et al., 1990). The participants were instructed to rate their memory on a 7-point Likert scale where 1 indicated severe problems, and 7 indicated no problems. These data were not analysed in the present study.

### Strategy Coding

Two independent judges (the first and the second authors) went through the participants’ strategy reports by applying a coding scheme. The coding instructions and concrete examples are provided in the Supplemental Table S1. The judges coded each strategy report for three variables: the first mentioned primary strategy type, the total number of different primary strategies used, and the number of strategy details mentioned. Inter-rater reliability for the strategy coding in the final sample was assessed by unweighted kappa (κ) for the first reported strategy type and with linearly weighted kappa (κw) for the other two strategy variables. The kappa coefficients suggested excellent agreement between the two raters for the first reported strategy type (κ = .89) and for the total number of strategy types (κw = .86), and substantial agreement for the total number of strategy details (κw = .76). Differences in coding were solved by subsequent consensus meetings. For the statistical analyses, we recoded the primary strategy types into three main types that reflect the level of strategy sophistication: no strategy use (NS), maintenance (MNT; rehearsal/repetition, selective focus), and manipulation (MNP; entailing manipulation of the memoranda in mind in one way or another: grouping, association, visualisation, narrative, condensing information). The remaining heterogeneous category of other strategies had only a few observations and was left out of the statistical analyses that involved the three main strategy types.

### Data Analysis

In accordance with our preregistered selection of Bayesian methods, we performed the statistical analyses with R version 4.4.0 employing the brms package (Bürkner, 2017). Bayes factor (BF) depicts the ratio of the likelihood of one hypothesis to the likelihood of the other, supporting either the experimental hypothesis over the null hypothesis (BF >1) or the null hypothesis over the experimental hypothesis (BF <1). BF values are expressed on a continuous scale with a range of 0–∞, where BF = 1 signals perfect ambiguity (the evidence favours neither hypothesis). All BFs reported here quantify evidence for the alternative hypothesis over the null hypothesis, and thus in the results, they are labelled as BF_10._ Our interpretation of BF values supporting either the experimental hypothesis (the BF_10_ values >1) or the null hypothesis (lack of an effect; the BF values <1) followed the descriptive guidelines by Kass and Raftery (1995). Thus, concerning the experimental hypothesis, BFs over 1 but not exceeding 3 constitute inconclusive (weak) evidence, BFs between 3 and 20 constitute positive evidence, BFs between 20 and 150 constitute strong evidence, and BFs over 150 constitute very strong evidence.

For all analyses, we used Bayesian mixed-effect models. The main analyses on word recall were conducted at the trial level, that is, the dependent variable was the correctness of recall (correct/incorrect) on all presented words per participant. With 207 participants and 10 words per block in 4 blocks, this totalled 8,280 individual trials. For research questions on other dependent variables, analyses could be at the block level or participant level.

Bayesian logistic mixed-effects regression (Bernoulli link function) was used to analyse the effects of the independent variables block and strategy on the correctness of word recall. We included block (within-subjects) as a fixed effect and varied the further predictors depending on the research question. We report BFs for the inclusion of main effects and interactions in the models and their respective coefficients as odds ratios (OR). We included a random intercept for each participant to account for baseline differences in level of memory performance and random slopes for block (treated as a categorical variable) to account for individual learning trajectories. We also included a random intercept for each presented word to account for potential differences in word saliency.

All models were run on 4 chains of Markov chain Monte Carlo simulations with 15,000 iterations (including 2,500 warmup iterations). Therefore, each model included 4 × 12,500 = 50,000 proper iterations. This number was determined by increasing the total number of iterations until all warnings of model instability were resolved.

Prior distribution for all parameters in brms models used the default Cauchy distribution with a scaling coefficient of .707 centred on zero as the prior distribution. This corresponds to a probability of 50% that the effect size lies between −0.707 and 0.707. The use of the default instead of more informative priors was motivated by the lack of previous empirical evidence on the effects of spontaneous memory strategy employment under PI. The two PI-related earlier strategy studies by Bass and Oswald (2014) and Massen and Vaterrodt-Plünnecke (2006) cited in the Introduction, focused on a specific externally provided strategy type, the method of loci, not on spontaneous strategies. As a complement, the main analyses for the critical PI blocks where word recall was the dependent variable were also conducted using extra thin and extra wide priors (corresponding to scale parameters 0.354 and 1.414). The results of these sensitivity analyses that probe the robustness of the observed effects are reported in the Supplemental Tables S5 to S7.

BFs were computed based on model comparison. For main effects, a model with both main effects present (but no interactions) was compared against a model with the evaluated independent variable omitted. In the case of interactions, a model with both main effects and their interaction was compared against a model with main effects only. Thus, for each “complete” analysis, four separate models were run: (a) the full model, (b) the main effects only model, (c) the main effect of block only model, and (d) the main effect of strategy variable only model. We report very large and very small BFs (over 1,000 and under 0.001) using exponential notation; for example, 1.000 × 10^3^ or 1.000 × 10^−3^. From the posterior distributions, we report as unstandardised effect sizes (odds ratios for recall correctness) accompanied by the 95% highest density credible intervals (HDI; values making up the middle 95% of the corresponding posterior distribution). ORs tell how much the likelihood of correct word recall changes depending on the independent variable. ORs over 1 indicate better recall, and ORs less than 1 indicate worse recall.

We tested the two preregistered hypotheses. Hypothesis 1 predicts a main effect for the group with strategy users exhibiting better word recall and fewer intrusion errors, but no group × block interaction. In other words, the performance difference between strategy users and no users should be more or less similar through the blocks, with both groups’ recall performance going down over the PI blocks. Hypothesis 2 predicts that in addition to the abovementioned main effect of group, the performance gap between strategy users versus non-users increases when PI builds up for blocks 2 and 3, leading to a group × block (blocks 1–3) interaction. It is worth noting here that strategy users and non-users are not the same participants throughout the blocks but vary depending on the strategic choices of individuals for each block. Thus, NS, MNT, or MNP strategy users in, say, block 1 are not identical with the NS, MNT, or MNP strategy users in block 2. This is taken into account in our statistical models, but not in blockwise mean values involving strategy variables, which mix within- and between-subject variability.

Besides the hypothesis testing described above, we compiled descriptive data on strategy use in the blocks, examined the frequency of strategy users in the blocks, and analysed possible differences in the number of strategy changers between the blocks. The last analyses concerned the three block transitions present in our setup, two within the PI buildup phase (PI1–PI2, PI2–PI3) and the third when moving to PI release (PI3–NEW).

## Results

### Descriptive Data

Table 2 depicts the distributions of strategy types in the four task blocks, both for the nine primary strategy types and the three main types of no strategy, maintenance, and manipulation. In all blocks, the majority of participants reported strategy use, with rehearsal/repetition being the most common primary strategy type. The absolute mean values of correctly recalled words per block for the main strategy types are given in the Supplemental Table S3.

**Table 2.** Distributions of the Nine Primary Strategies and the Three Main Strategy Types (No Strategy, Maintenance, Manipulation) per Block.

Strategy	Frequency (%)			
	PI1	PI2	PI3	NEW
No strategy	54 (26.1)	52 (25.1)	56 (27.1)	52 (25.1)
Maintenance	79 (38.2)	82 (39.6)	77 (37.2)	68 (32.9)
Rehearsal/repetition	76 (36.7)	79 (38.2)	73 (35.3)	62 (30)
Selective focus	3 (1.4)	3 (1.4)	4 (1.9)	6 (2.9)
Manipulation	72 (34.8)	69 (33.3)	67 (32.4)	83 (40.1)
Grouping	13 (6.3)	16 (7.7)	18 (8.7)	25 (12.1)
Association	17 (8.2)	17 (8.2)	19 (9.2)	29 (14)
Visualisation	22 (10.6)	20 (9.7)	18 (8.7)	20 (9.7)
Narrative	14 (6.8)	10 (4.8)	9 (4.3)	5 (2.4)
Condensing information	6 (2.9)	6 (2.9)	3 (1.4)	4 (1.9)
Other	2 (1)	4 (1.9)	7 (3.4)	4 (1.9)

*Note.* PI1–3 = the proactive interference blocks. NEW = the fourth block with new items that provided release from proactive interference.

For those participants who employed a strategy, Table 3 shows the level of strategy detail (LoD) and the number of strategies reported as a function of block and main strategy type. Overall, zero details (i.e., just describing the strategy without giving any concrete details like “I created a story of the words”) were the most common LoD value, but manipulation strategy users reported concrete details of their strategy more often than maintenance strategy users did. Regarding the number of primary strategies, the overwhelming majority of strategy users reported only a single strategy for each block.

**Table 3.** Distributions of Strategy Level of Detail and Number of Primary Strategies in Strategy Users per Main Strategy Type and Block.

Block	Strategy type	No details (%)	Details present (%)
LoD			
PI1	MNT	67 (83)	6 (7)
	MNP	29 (39)	28 (37)
PI2	MNT	74 (87)	9 (11)
	MNP	33 (46)	27 (38)
PI3	MNT	75 (90)	7 (8)
	MNP	33 (49)	21 (31)
New words	MNT	58 (82)	10 (14)
	MNP	19 (23)	43 (51)
Block	Strategy type	Single(%)	Multiple(%)
Number of strategies reported			
PI1	MNT	66 (84)	13 (16)
	MNP	57 (79)	15 (21)
PI2	MNT	66 (80)	16 (20)
	MNP	52 (75)	17 (25)
PI3	MNT	63 (82)	14 (18)
	MNP	52 (78)	15 (22)
New words	MNT	56 (82)	12 (18)
	MNP	68 (82)	15 (18)

*Note.* PI1–3 = the proactive interference blocks. NEW = the fourth block with release from proactive interference. MNT = maintenance strategy users; MNP = manipulation strategy users; LoD = level of strategy detail.

Descriptive data on intrusion errors are given in Supplemental Table S4. The absolute values were very low, albeit hinting at an increase from block 2 to block 3, as one would expect under accumulating PI. Due to their scarcity, intrusion errors were omitted from the analyses.

We examined block-level data to determine whether the subjective task difficulty ratings participants gave after each block changed across the experiment. A Bayesian mixed model (Gaussian link) with block as a predictor and random intercepts included for each participant showed very strong evidence for the effect of block, both for all blocks (BF_10_ = 4.223 × 10^13^) and for the PI blocks only (BF_10_ = 3.292 × 10^14^). As one would expect, average subjective difficulty ratings in Figure 2 suggest some increase during the PI blocks 1 to 3, followed by a decrease to the initial levels with release from PI in the final block.

**Figure 2.**
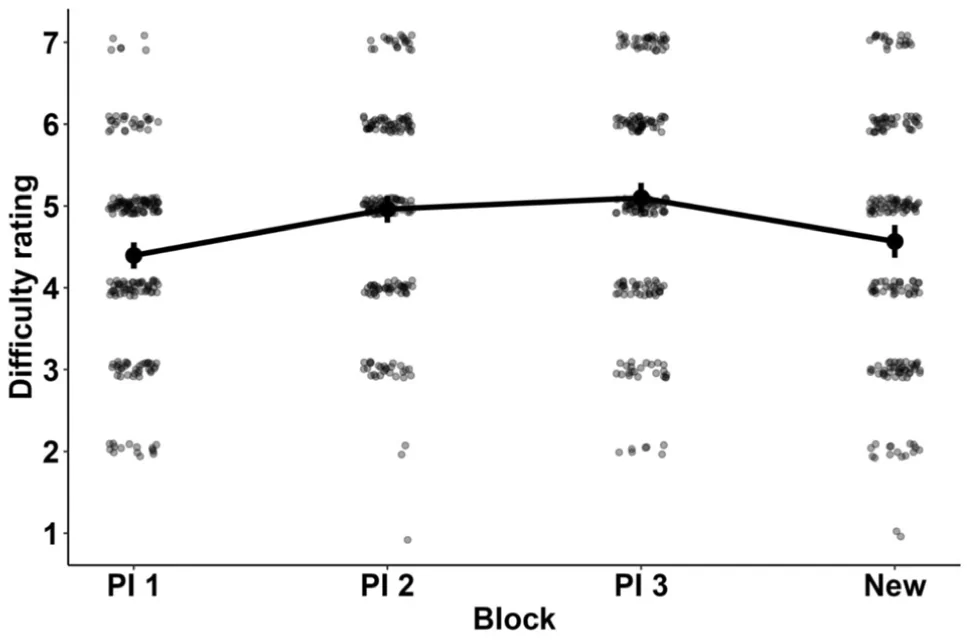
Subjective difficulty ratings across the task blocks.

### Strategy Type and Word Recall

We used logistic (Bernoulli link function) Bayesian mixed models to examine the effects of block and main strategy type (no strategy, maintenance strategy, manipulation strategy) on word recall (Table 4 and Figure 3). The analysis was done on a trial level. A random intercept and a random slope for block were specified for each participant, and a random intercept for each presented word. When all four blocks were considered, there was very strong evidence for the main effects of block (BF_10_ = 1.728 × 10^8^) and strategy type (BF_10_ = 2.064 × 10^7^), and very strong evidence against their interaction (BF_10_ = 9.378 × 10^−5^). The same pattern was also observed when only the PI blocks 1 to 3 were included. Compared to block PI1, odds of correctly recalling words were 23% lower in block PI2 (OR = 0.77, 95% HDI [0.61, 0.98]) and 39% lower in block PI3 (OR = 0.61, 95% HDI [0.48, 0.78]), indicating the presence of proactive interference. Supporting this, there was almost no change in word recall performance between the first PI block and the novel word list in block 4 (OR = 0.96, 95% HDI [0.72, 1.26]). Contrast analyses from the full model revealed that compared to no strategy, odds of correct word recall were 42% higher with maintenance strategy (OR = 1.42, 95% HDI [1.17, 1.67]) and 81% higher recall with manipulation strategy; MNP: OR = 1.81, 95% HDI [1.51, 2.14]). Moreover, manipulation strategies resulted in 28% higher odds of correct recall when compared against maintenance strategies (OR = 1.28, 95% HDI [1.07, 1.49]).

**Table 4.** Relationships Between Main Strategy Type (No Strategy, Maintenance, Manipulation) and Word Recall Performance.

Model term	All blocks, OR [95% HDI]	PI blocks only, OR [95% HDI]
Intercept	1.82 [1.45, 2.28]	1.82 [1.45, 2.30]
Block	BF_10_ = 1.728 ⨯ 10^9^	BF_10_ = 1.815 ⨯ 10^8^
PI2	0.77 [0.61, 0.98]	0.78 [0.61, 0.99]
PI3	0.61 [0.48, 0.78]	0.61 [0.47, 0.78]
NEW	0.96 [0.72, 1.26]	—
Strategy type	BF_10_ = 2.064 ⨯ 10^7^	BF_10_ = 2.848 ⨯ 10^4^
MNT	1.46 [1.13, 1.87]	1.44 [1.11, 1.87]
MNP	1.77 [1.37, 2.30]	1.73 [1.33, 2.27]
Block ⨯ strategy type	BF_10_ = 9.374 ⨯ 10^−4^	BF_10_ = 0.001
PI2 ⨯ MNT	0.96 [0.70, 1.32]	0.97 [0.70, 1.34]
PI3 ⨯ MNT	0.88 [0.64, 1.21]	0.90 [0.65, 1.26]
NEW ⨯ MNT	1.07 [0.77, 1.49]	—
PI2 ⨯ MNP	0.94 [0.68, 1.31]	0.92 [0.66, 1.29]
PI3 ⨯ MNP	1.05 [0.75, 1.47]	1.06 [0.75, 1.49]
NEW ⨯ MNP	1.10 [0.79, 1.54]	—

*Note.* OR values over 1 signify better recall, and values under 1 worse recall. Coefficients are from full models. Reference categories are block PI1 and no strategy use. BFs quantify evidence for (BF >1) or against (BF <1) the presence of the main effects and the interaction. OR = odds ratio; BFs = Bayes factors; MNT = maintenance strategy users; MNP = manipulation strategy users; HDI = highest density credible intervals.

**Figure 3.**
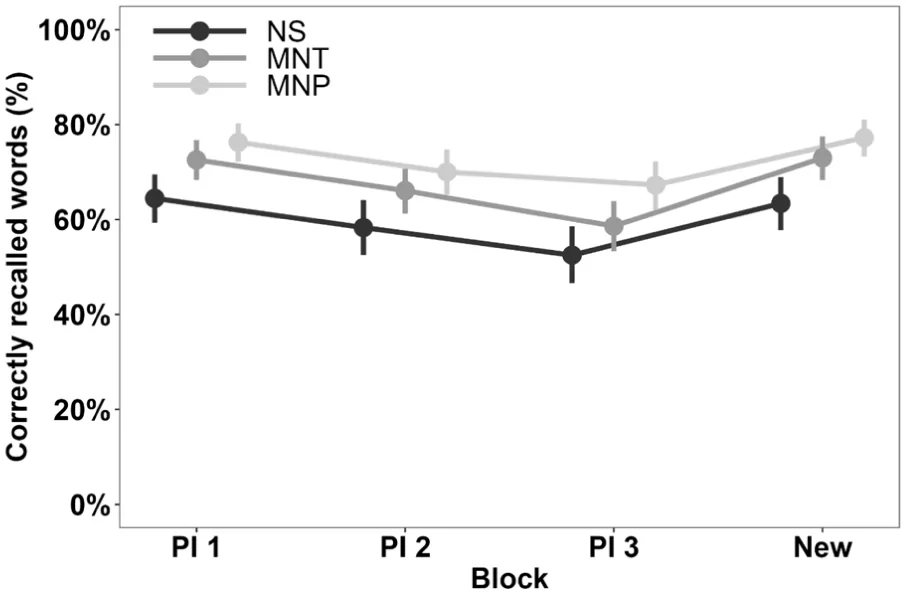
Proportion of correctly recalled words in participants reporting no strategy, maintenance strategy, or manipulation strategy for that task block.

### LoD and Word Recall

For these analyses, we included trials only from those blocks where a participant reported having used a strategy. In this analysis, LoD was treated as a dichotomised variable with either none (0) or some (1 or more) strategy details present. The main effects of LoD, block, and their interaction were examined with a logistic Bayesian mixed model (Table 5 and Figure 4). The analysis was done at the trial level, and the random effect structure was the same as in the previous model. As expected, results with the block variable were very similar to the previous analysis, so we do not cover them in detail again here or in the next section. We obtained positive evidence for the effect of LoD in all trials in complete data with all four blocks (BF_10_ = 5.86, OR = 1.22, 95% HDI [0.96, 1.58], and strong evidence when only the three PI blocks were considered (BF_10_ = 95.41, OR = 1.32, 95% HDI [1.02, 1.71]), indicating that those with more detailed strategy reports had better recall. We also found moderate evidence for the lack of block × LoD interaction in both complete data covering all four blocks (BF_10_ = 0.11) and in the PI blocks only (BF_10_ = 0.18), meaning the effect of strategy detail did not change between the blocks.

**Table 5.** Relationships Between Level of Strategy Detail and Word Recall Performance.

Model term	All blocks, OR [95% HDI]	PI blocks only, OR [95% HDI]
Intercept	2.71 [2.21, 3.33]	2.60 [2.13, 3.20]
Block	BF_10_ = 3.764 ⨯ 10^5^	BF_10_ = 2.356 ⨯ 10^4^
PI2	0.75 [0.61, 0.91]	0.76 [0.62, 0.93]
PI3	0.54 [0.44, 0.66]	0.55 [0.45, 0.68]
NEW	1.08 [0.83, 1.41]	—
Level of detail	BF_10_ = 5.856	BF_10_ = 95.405
Details present	1.22 [0.96, 1.58]	1.32 [1.02, 1.71]
Block ⨯ level of detail	BF_10_ = 0.112	BF_10_ = 0.180
PI2 ⨯ details present	1.00 [0.71, 1.41]	0.99 [0.69, 1.40]
PI3 ⨯ details present	1.40 [0.97, 2.03]	1.37 [0.94, 2.02]
NEW ⨯ details present	0.92 [0.66, 1.28]	—

*Note.* OR values over 1 signify better recall, and values under 1 worse recall. Coefficients are from full models. Reference categories are block PI1 and no strategy details present. BFs quantify evidence for (BF >1) or against (BF <1) the presence of the main effects and the interaction. OR = odds ratio; BFs = Bayes factors; PI = proactive interference; HDI = highest density credible intervals.

**Figure 4.**
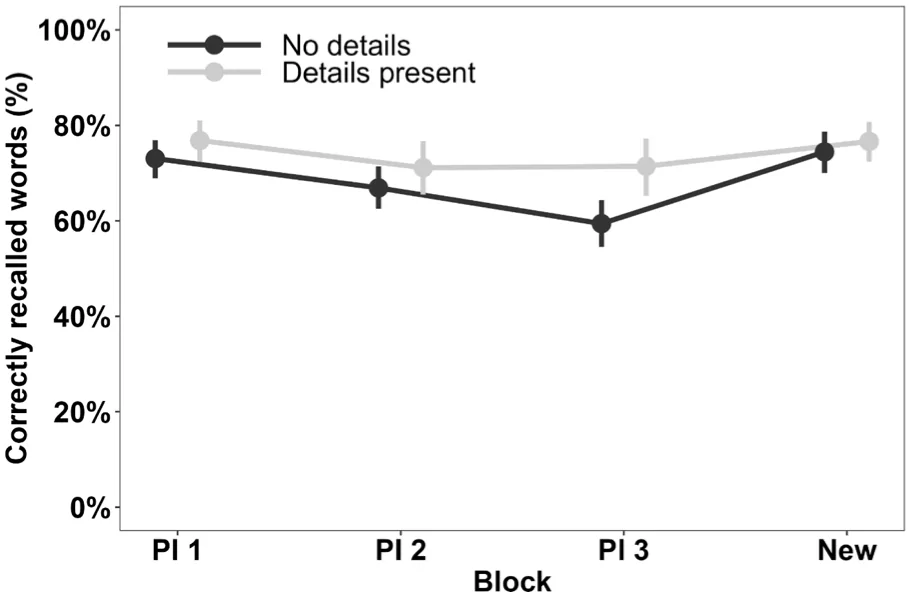
Blockwise recall performance in strategy users as a function of the level of strategy detail they reported.

### Number of Primary Strategies and Word Recall

We analysed the number of strategies reported in the same way as LoD (Table 6 and Figure 5). Again, only trials in blocks where strategy use was reported were included in the analysis. The number of strategies variable was dichotomised into single strategy versus multiple strategy use. The analysis was done on the trial level, and the model specification was the same as before, but with the number of strategies as an independent variable besides block. The evidence for the effect of multiple strategies was inconclusive in complete data covering all four blocks (BF_10_ = 1.15, OR = 1.44, 95% HDI [1.06, 2.00]) and positive when only the three PI blocks were considered (BF_10_ = 5.52, OR = 1.55, 95% HDI [1.12, 2.17]), suggesting that using multiple strategies is more beneficial for recall under PI. We also obtained moderate to strong evidence for the lack of interaction between block and the number of strategies in all blocks (BF_10_ = 0.02) and in the PI blocks (BF_10_ = 0.08).

**Table 6.** Relationships Between the Number of Primary Strategies and Word Recall Performance.

Model term	All blocks, OR [95% HDI]	PI blocks only, OR [95% HDI]
Intercept	2.76 [2.29, 3.35]	2.68 [2.22, 3.25]
Block	BF_10_ = 3.471 ⨯ 10^6^	BF_10_ = 8.128 ⨯ 10^4^
PI2	0.76 [0.63, 0.92]	0.77 [0.64, 0.93]
PI3	0.59 [0.49, 0.71]	0.60 [0.49, 0.73]
NEW	1.09 [0.86, 1.38]	—
Number of strategies	BF_10_ = 1.147	BF_10_ = 5.516
Multiple strategies	1.44 [1.06, 2.00]	1.55 [1.12, 2.17]
Block ⨯ number of strategies	BF_10_ = 0.017	BF_10_ = 0.084
PI2 ⨯ multiple strategies	0.78 [0.52, 1.17]	0.77 [0.51, 1.17]
PI3 ⨯ multiple strategies	0.87 [0.57, 1.32]	0.88 [0.57, 1.35]
NEW ⨯ multiple strategies	0.86 [0.55, 1.33]	—

*Note.* OR values over 1 signify better recall, and values under 1 worse recall. Coefficients are from full models. Reference categories are block PI1 and single strategy used. BFs quantify evidence for (BF >1) or against (BF <1) the presence of the main effects and the interaction. OR = odds ratio; BFs = Bayes factors; PI = proactive interference; HDI = highest density credible intervals.

**Figure 5.**
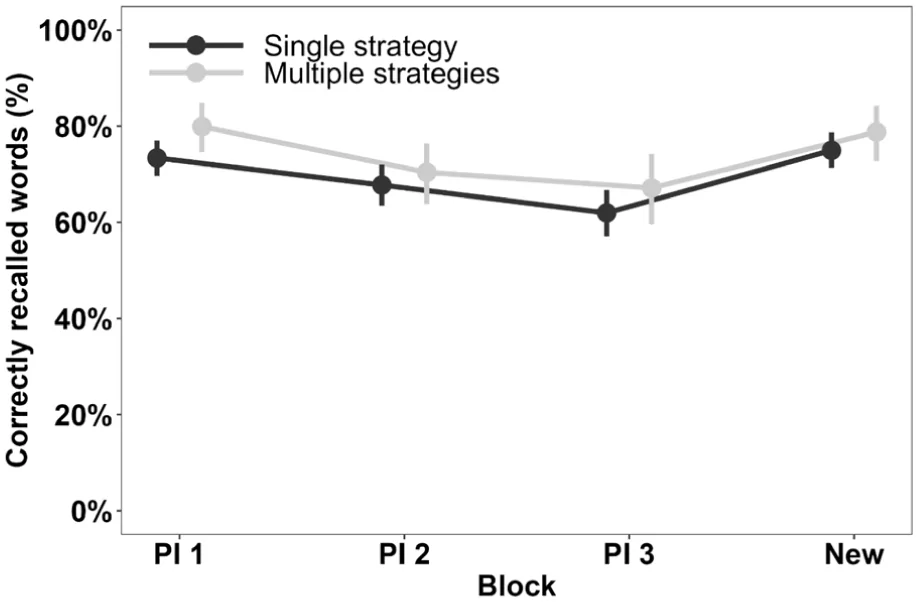
Blockwise recall performance in strategy users as a function of the number of primary strategies (single, multiple) employed.

### Strategy Development

We also examined whether strategy employment changed across the task blocks. Dummy-coded main strategy type, LoD, and the number of reported strategies were all entered as dependent variables in their own models with block as the sole predictor and participant intercept as the sole random effect. These analyses were done at the block level, so we did not include block as a random slope for each participant, as this would have identified every data point.

Dummy-coded strategy types were examined with logistic Bayesian mixed models. When all four blocks were considered, there was positive-to-weak evidence against the effect of block for the rate of no strategy (BF_10_ = 0.032), maintenance strategy (BF_10_ = 0.253), and manipulation strategy (BF_10_ = 0.396). This also applied when only the three PI blocks were considered (NS: BF_10_ = 0.112, MNT: BF_10_ = 0.114, MNP: BF_10_ = 0.107). These results imply that on the aggregate level, there were no major shifts in strategy type usage across the blocks.

LoD and the number of strategies, being count-type variables, were analysed using Bayesian mixed models (random intercepts for each participant) with a Poisson-type link function. With the LoD, we found strong evidence for the main effect of block (BF_10_ = 851.92), indicating that the LoD decreased slightly as the PI phase progressed, and increased again in the new word list (see Figure 6A). However, when the new word list block was left out of the analyses, the evidence was towards no effect (BF_10_ = 0.60). In turn, for the number of strategies (Figure 6B), there was strong to very strong evidence against the main effect of block for both the complete data with all four blocks (BF_10_ = 0.001) and for the three PI blocks (BF_10_ = 0.012), showing that the number of strategies reported remained constant across the blocks.

**Figure 6.**
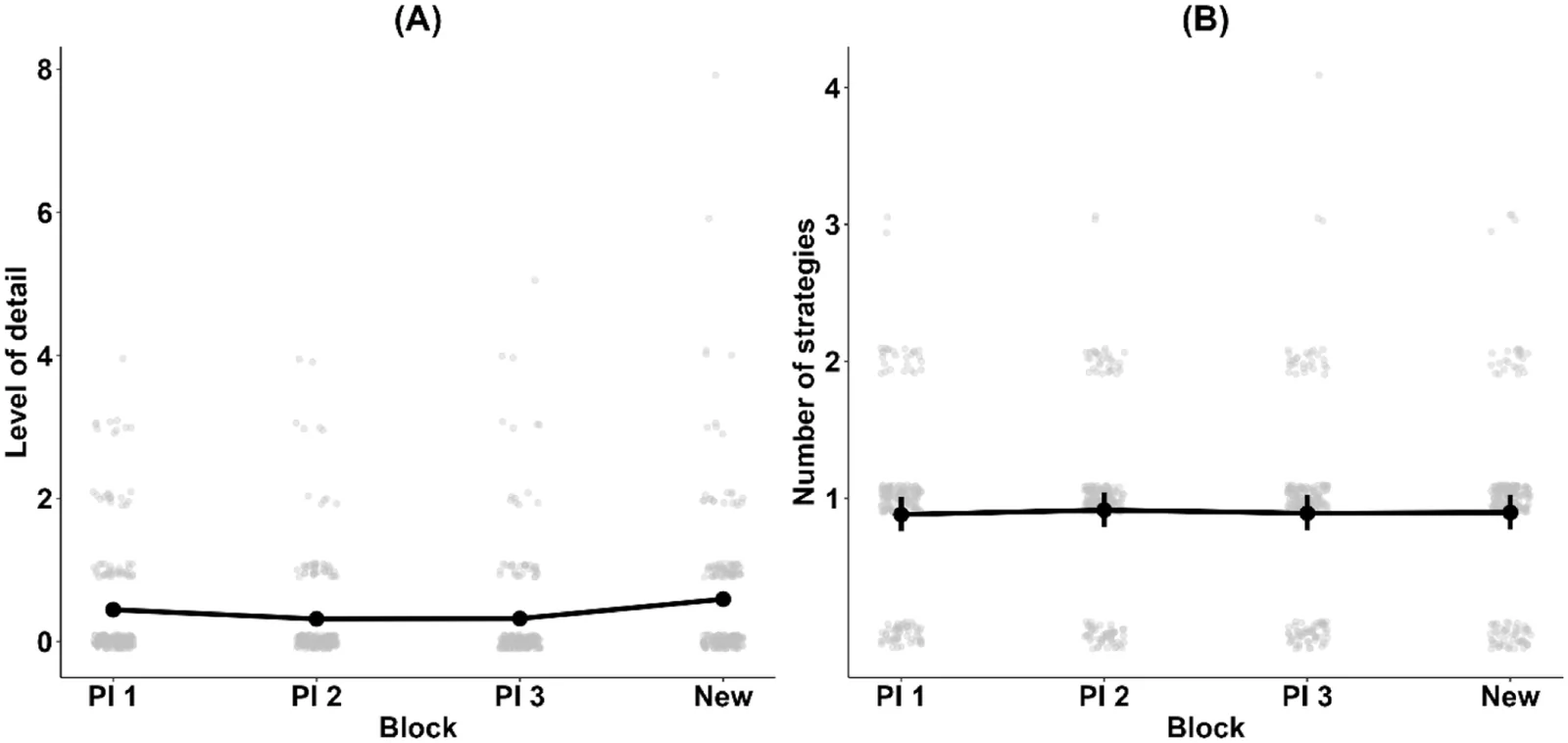
(A) Level of strategy detail and (B) number of primary strategies reported in each task block.

### Strategy Use and Change of Primary Strategy Across the Blocks

Finally, we analysed the rates of strategy use/no use and strategy changes between the blocks separately for all blocks and for the three PI blocks only. For strategy use, binary strategy usage (yes/no) was entered as a dependent variable into a logistic Bayesian mixed model with block as the sole predictor (Figure 7A). The random effect structure only included random intercepts for each participant. We conducted the same analysis with and without the last release from the PI block. When all four blocks were included, we found very strong evidence against the main effect of block for strategy use (BF_10_ = 0.036) (see Figure 7A). Strong evidence for the absence of the main effect of block was also obtained when considering only PI blocks (BF_10_ = 0.119).

**Figure 7.**
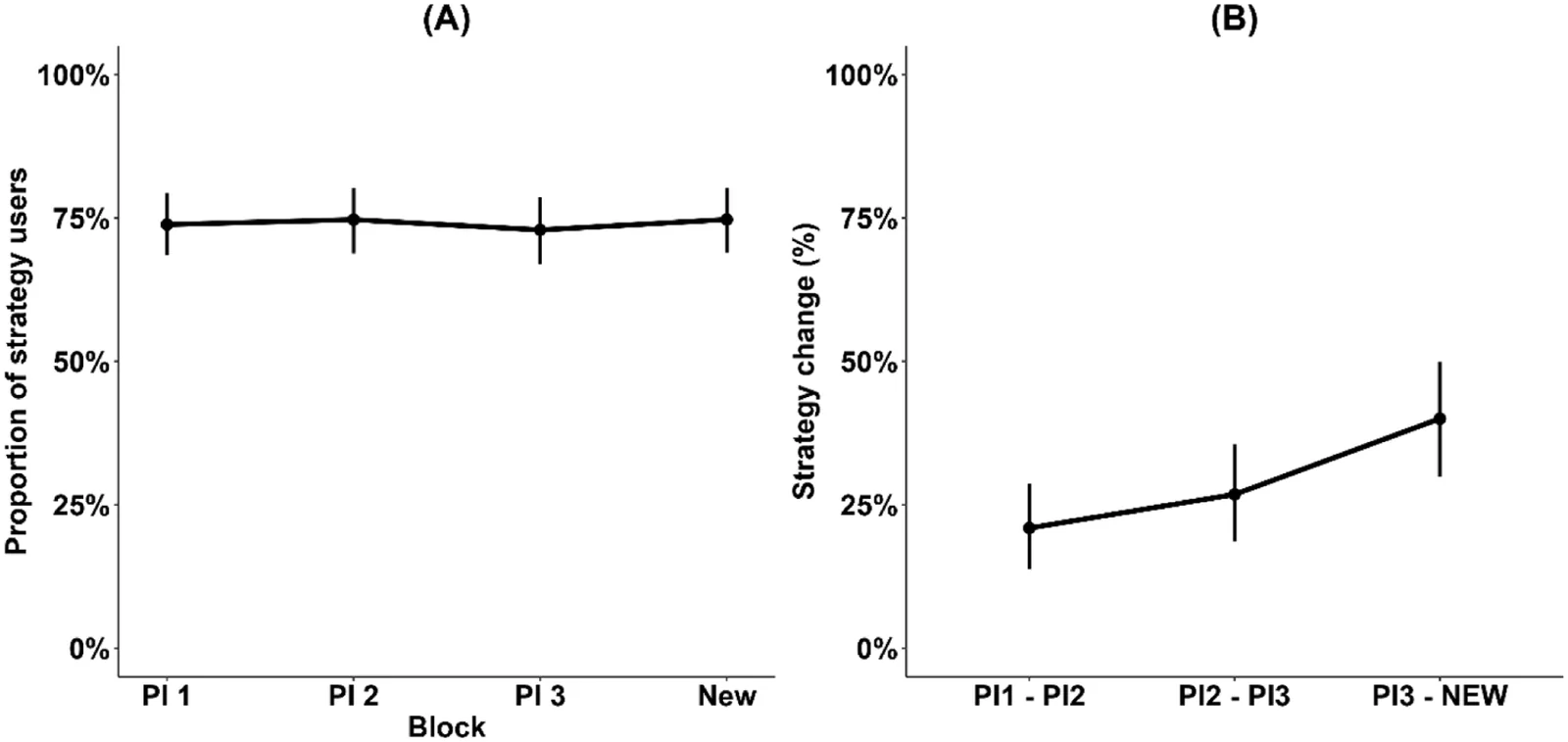
(A) Rates of strategy users versus non-users in each block and (B) strategy changers between the task blocks.

To analyse the rates of strategy changers (Figure 7B), we focused on the transitions between successive blocks. Here, a unit of analysis was a transition between blocks, and dependent variable a binary variable indicating whether a strategy change occurred or not. We used Bayesian mixed models (Bernoulli link) with block as the sole predictor and random intercepts for each participant. When all four blocks were examined, there was strong evidence for the main effect of block shift (BF_10_ = 93.02) with a higher percentage of strategy switching in later blocks. However, when the transitions between the first three PI blocks (PI1–PI2, PI2–PI3) were examined, there was only inconclusive evidence against the effect of block shift (BF_10_ = 0.841).

Regarding the direction of change of the main strategy type when moving from the last PI block to release from PI, 38 participants changed to a more advanced strategy (from no strategy to maintenance/manipulation or from maintenance to manipulation) and 22 to a less advanced strategy. Evidence for an asymmetry in this direction of change was inconclusive in a Bayesian binomial test (BF_10_ = 1.335).

## Discussion

This preregistered study examined the role of spontaneous mnemonic strategy use in categorised word list recall under increasing PI. We formulated and tested two hypotheses. Both hypotheses assume better word recall in strategy users but are distinct in their predictions concerning performance differences during accumulating PI. The first hypothesis states that under PI, performance goes down at the same rate in strategy users and non-users, thus showing a main effect of group but no group × block interaction. In turn, the second hypothesis assumes that the performance curves of strategy users and non-users diverge over the PI blocks so that strategy non-users exhibit a more marked performance decrease over the PI blocks. This should lead to a group × block interaction, besides the main effect of group. The results showed that most participants reported strategy use, and the proportion of strategy users remained stable across the blocks, being roughly evenly distributed between maintenance and manipulation strategy users. As to our two competing hypotheses, the results provided support for the first one. While recall performances dropped through the first three PI blocks, the advantage of strategy users – best recall for manipulation strategy users, followed by maintenance strategy users and no strategy users – did not change across the three PI blocks, as all three groups exhibited a similar decline in recall performance.

Our results suggest that in a PI setting, spontaneous strategy use in word list recall is beneficial to memory performance. This is in line with earlier results concerning externally provided mnemonic strategies under PI (Bass & Oswald, 2014; Massen & Vaterrodt-Plünnecke, 2006), as well as other strategy-related research employing a variety of non-PI learning and memory task paradigms (Dunlosky & Hertzog, 2001; Dunlosky & Kane, 2007; Laine et al., 2018, 2024b; McNamara & Scott, 2001; Reese-Melancon et al., 2019; Unsworth et al., 2019; Waris et al., 2021a, 2021b). Moreover, the level of strategy sophistication was associated with recall performance, with manipulation strategy users showing the highest recall. Similar findings on the superiority of manipulation strategies have also been obtained in earlier studies of word list learning (Laine et al., 2024a; Waris et al., 2021a), concurring with the classical levels of processing view that posits better recall for items that have been elaborated and not merely repeated during encoding (Craik & Lockhart, 1972).

While being beneficial for recall, the present results indicate that the use of spontaneous strategies does not specifically guard against accumulating PI. As noted in the Introduction, the rise of PI has been related to both encoding and recall. In encoding, diminishing attentional resources over time have been postulated (Engle, 1975; Johansson et al., 2018; Kane & Engle, 2000), while retrieval accounts of PI emphasise more diffuse search sets where an increasing number of items share the same category-based retrieval cues (Watkins & Watkins, 1976; Wixted & Rohrer, 1993). The spontaneous strategies adopted by our participants enhanced recall throughout the task but did not appear to provide enough differentiation between current and previous category members to prevent or slow down the buildup of PI. To pick up some concrete examples from the reports involving different manipulation strategies, memorising “the first letters of the animals” (condensing information) used by one participant would not abolish interference, as some animal names shared the same initial letters. Another participant “tried to create a visual sequence of each animal interacting with the next animal” (visualisation of objects and/or locations) but added that “because animals are similar things and the list went by quite quickly with no chance to rehearse it, the mnemonic didn’t really work!.” A third participant “tried to group the animals in my mind and think of them from the country they belong to” (grouping), but this strategy could also be affected by cumulative interference as the lists of animal names were heterogeneous and not country specific. The success of trying “to create story using the animals in the order they appeared” (narrative) by a fourth participant would depend on the distinctiveness of the story from previous ones.

Certain advanced mnemonic strategies, such as a variant of the classical method of loci, with word lists mentally located in separate rooms and items in specific locations in those rooms, can significantly attenuate PI (Bass & Oswald, 2014). However, it is highly unlikely for a naive participant to spontaneously develop and employ such a mnemonic system. Only one of our participants “tried imagining myself walking through my house and encountering the animals in different parts of the house” (visualisation of objects and/or locations), but added that “it didn’t really work,” perhaps due to lack of practice with this classical mnemonic technique. Thus, in the next PI block, this participant gave up with the method of loci and “just kept repeating the words in my head” (rehearsal/repetition).

Interestingly, we saw evidence for an increase in strategy changers when our participants moved from the last PI block to release from PI. This result is notable as several previous blockwise strategy studies on memory and learning have reported the opposite pattern of gradual strategy stabilisation across task blocks (Laine et al., 2024a, 2025; Waris et al., 2021a, 2021b). It seems plausible that this discrepancy stems from the nature of the memory tasks employed. In these previous studies, the same to-be-learned stimulus list was presented over several blocks (Laine et al., 2024a, 2025; Waris et al., 2021a), or the researchers employed a memory updating task where the difficulty level was matched with the participant’s learning rate (Waris et al., 2021b). In contrast, the release from PI in the present setup can be seen as a qualitative change in the task that prompted alternative strategy choices in part of the participants. This interpretation finds support also in the blockwise subjective difficulty ratings which go up for the successive PI blocks, and then down for release from PI in the final block (see Figure 2).

We also examined two other aspects of spontaneous strategy in strategy users. Both strategy level of detail and the number of strategies employed were associated with better recall, albeit the evidence was weaker and less stable for the latter feature (see also the sensitivity analyses in the Supplemental Material). Two previous studies using repetitive word list learning also found an advantage in recall with higher LoD (Laine et al., 2024a; Waris et al., 2021a). However, in the present study, the effect of the strategy level of detail on recall should be taken with caution, as details were most frequent amongst the manipulation strategy users.

Several limitations of the study are worth noting. First, as we examined self-generated strategies, the present evidence for the beneficial effects of strategy use on recall under PI is correlative. However, the studies on enhanced memory performance with externally provided memory strategies under PI discussed earlier speak for a causal effect of strategy use (Bass & Oswald, 2014; Massen & Vaterrodt-Plünnecke, 2006). Further evidence for the validity of subjective strategy reports could be obtained by trying to relate them to objective strategy measures. For example, in word list learning, a detailed grouping strategy (e.g., “I memorised the words in chunks – domestic animals, birds, and fish”), if valid, should also be verifiable in a corresponding clustering in the order in which the participant recalls the items. For a discussion on objective clustering indices derived from the recall pattern in word list learning, see Stricker et al. (2002).

Second limitation is that introspective reports may provide an incomplete picture of strategy use. For example, one could claim that the quality and detailedness of subjective strategy reports merely reflect general task engagement. While it is evident that some engagement is necessary to perform any cognitive task, an earlier study on spontaneous strategy use in a working memory task by Waris et al. (2021b) found that strategy level of detail showed rather weak correlations with self-estimated task motivation (*r* = .173; shared variance ca 3%) and with self-estimated effort put on the task (*r* = .240; shared variance ca 6%). In a similar memory strategy study employing word list learning, evidence for an effect of motivation on memory strategy measures was either weak or supported the null hypothesis with weak or positive evidence (Waris et al., 2021a). Waris et al. (2021b) also probed the association between verbosity in a picture description task and strategy level of detail in their working memory task, finding some overlap between these two variables (*r* = .323; shared variance ca 10%). Thus, strategy reports may also partly reflect the verbal skills of a participant.

The third limitation is the possible effect of repeated reporting of strategies instead of the more common use of a single post-task report on strategy use. The word list learning study by Waris et al. (2021a) addressed this issue by comparing a group that gave open-ended strategy reports after each of the five task blocks to another group that reported their strategies only after the final, fifth task block. They found no difference in objective recall, but those who had repeatedly filled out the open-ended strategy reports showed somewhat less strategy sophistication and detail on the final task block. Waris et al. (2021a) speculated that this could reflect some degree of saturation and weariness due to repeated reporting. One could note here that, in contrast to open-ended strategy reports, repeated list-based strategy queries do appear to bias both strategy use and memory performance (Waris et al., 2021b). Thus, open-ended strategy reports, while being more tedious to analyse, appear more suitable for repeated use to examine possible within-task strategy evolvement, with the caveat that some weariness of reporting may occur across the task blocks.

The fourth limitation is related to the online nature of the study. We could not control the testing situation, albeit our anonymous participants were instructed to find a distraction-free place and occasion. In previous comparative studies, the results from lab-based versus web-based cognitive testing have been quite comparable, with the possible exception of reaction times that were not the focus here (e.g., Backx et al., 2020; Germine et al., 2012).

In conclusion, the present study sheds light on the hitherto unstudied relationships between spontaneous mnemonic strategies and memory performance under PI. Our results highlight the overall benefit of strategy use and reveal flexible strategy selection after a qualitative change in task demands. At the same time, the strategies our participants spontaneously generated did not prevent the accumulating deleterious effects of PI.

## Supplemental Material

sj-docx-1-qjp-10.1177_17470218261454265 – Supplemental material for Spontaneous Mnemonic Strategy Use Under Proactive Interference Is Related to Better Recall But Does Not Protect from Accumulating Interference Supplemental material, sj-docx-1-qjp-10.1177_17470218261454265 for Spontaneous Mnemonic Strategy Use Under Proactive Interference Is Related to Better Recall But Does Not Protect from Accumulating Interference by Matti Laine, Tilda Eräste and Anton Kunnari in Quarterly Journal of Experimental Psychology

## Data Availability

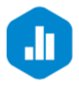 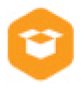 The data and materials from the present experiment are publicly available at the Open Science Framework website: https://osf.io/m7nx6/. The preregistration of the study can be found at https://aspredicted.org/4DZ_F5K.
