# Supplementary material for: Spontaneous Mnemonic Strategy Use Under Proactive Interference Is Related to Better Recall But Does Not Protect from Accumulating Interference
Source: Q J Exp Psychol (Hove). 2026 May 20;79(11):2991–3004. doi: 10.1177/17470218261454265 (PMC13675575; doi:10.1177/17470218261454265)
Supplement: Supplementary file 1 [file sj-docx-1-qjp-10.1177_17470218261454265.docx]

Supplementary Material for:

**Spontaneous mnemonic strategy use under proactive interference is related to better recall but does not protect from accumulating interference**

Matti Laine^1^, Tilda Eräste^1,2^ & Anton Kunnari^3^

^1^Department of Psychology, Åbo Akademi University, Finland

^2^Unit of Psychology, University of Oulu, Finland

^3^Department of Psychology and Logopedics, University of Helsinki, Finland

**Corresponding author**: Matti Laine, Department of Psychology, Åbo Akademi University, Fabriksgatan 2, 20500 Åbo, Finland. ORCID ID: 0000-0003-4529-4965. Email: [matti.laine@abo.fi](mailto:matti.laine@abo.fi)

**Supplementary Table S1. Strategy coding guidelines.**

**GENERAL INSTRUCTIONS FOR THE INDEPENDENT JUDGES TO CODE PARTICIPANTS’ OPEN-ENDED BLOCK-WISE MEMORY STRATEGY DESCRIPTIONS IN WORD LIST RECALL**

- For each strategy description, code three variables: the first reported strategy type (there can be only one primary strategy), the total number of strategy types reported, and the total number of specific strategy details given (be they for one or more strategy types).
- If the strategy description field is completely empty, do not code anything.
- If the participant has typed in “None” but nevertheless given a subsequent description of a strategy, evaluate the response from the strategy description.
- If the participant refers to a strategy s/he used in a previous task block, evaluate the response from the strategy description for that other block.
- If the participant refers to a strategy s/he used for another block that cannot be identified on the basis of the response, mark this as “other strategy”.
- Extraneous information not related to strategy use such as “it went bad”, “the task was difficult”, “my mom disturbed me during task performance” should not be taken into account when rating the strategy descriptions, neither comments that merely describe the sequence of actions one took when performing the task block, or comments that merely recount the task characteristics or the task instructions. Keep in mind that strategy is an identifiable, self-generated way or ways of managing a memory task, thus requiring activity from the part of the participant.
- Possible use of external aids (e.g., “wrote down what needed to be done”) are coded as 999, as these participants will be discarded from the strategy analyses.

**CODING CRITERIA: STRATEGY TYPE**

| **Code for strategy type** | **Type** | **Examples** |
| --- | --- | --- |
| **0** | **No explicit strategy use**   - No strategy - No clear evidence of strategy use - Repetition of instructions or own action sequence - Response unrelated to strategy use, comments on understanding instructions, or comments on task difficulty - Guessing, intuition, instinct, familiarity, ‘gut feeling’ | - “No system” - “I tried” - “I tried to keep up with the list but found it quite difficult” - “Just tried to concentrate on the words” - “I memorized the words mentally” - “I memorized all the words” - “Tried to apply a strategy/mnemonic”(with no description as to what the strategy/mnemonics would have been) |
| **1** | **Rehearsal / Repetition**   - Rehearsing items verbally, nonverbally or visuomotorically - *E.g. repeating, speaking in head, saying in mind, yelling the tasks, rehearsing quietly* | - “I repeated the words” - “I rehearsed the words in my mind” - “Said aloud all the items in the list in order to keep them in memory” |
| **2** | **Grouping**  - Grouping items together  - *E.g. chunking, pairing, memorizing in groups, remembering in chunks, thinking in pairs, dividing into subsets, subdividing into sets, splitting into shorter sequences* | - “I memorized the words in chunks” - “I grouped the words in pairs” - “I remembered the words in groups” - “grouped together in my mind words that rhymed” |
| **3** | **Association**  - Associating the objects/actions with something outside the stimuli | - “I tried to associate each word with something that I have at home” - “I associated the words to characters in different movies or cartoons” - “I tried to relate the words to my friends” |
| **4** | **Visualization of objects and/or locations**  - Linking tasks with some visual/visuospatial representations in mind  - *E.g. visualizing objects in mind, forming spatial images, visualizing objects in their locations* | - “Built a mental image of the words in my mind” - “Visualized the words as they were shown” - “I was picturing the words in my head” - “I tried imagining myself walking through my house and encountering the words in different parts of the house” |
| **5** | **Selective focus**   - Focusing on only some of the words | - “I focused on remembering only a subset of the words and disregarded the rest” - “Just concentrated on the easy words as the task was quite difficult” - “Fixated only on the first 4 words” |
| **6** | **Narrative**   - Using the words to form a story | - “Made up an anecdote based on the words to remember them better” - “I tried to create a story in my head that used the words in a sentence to try and help me remember them” |
| **7** | **Condensing information**   - Abbreviating the stimuli in one way or another | - “Tried to memorize the first syllables of the words” - “I tried to recall the first letters of each word” |
| **8** | **Other strategy**   - Describing a strategy that cannot be classified in any of the categories above | - “I tried to match the words to those I saw previously” - “Tried to do alphabetically” - “I tried to sing the words to make a song” - “I tried to remember in a rhyme” |

**CODING CRITERIA: STRATEGY LEVEL OF DETAIL**

| **Code for strategy level of detail** | **Level** | **Examples** |
| --- | --- | --- |
| **0** | **No strategy use**   - No strategy employed - Vague comments that do not point to an identifiable strategy | - “No system” - “I tried” - “I tried to keep up but found it quite difficult” - “Just tried to concentrate on the words” - “I memorized the words mentally” - “I memorized all the words” - “Tried to apply a strategy/mnemonic” |
| **0** | **An identifiable strategy or strategies without any specific detail** | - “Grouped the words to help to remember them” *(grouping)* - “I rehearsed the words” *(rehearsal)* - “I repeated the words as they came, and then divided them into groups” *(rehearsal, grouping)* |
| **1** | **An identifiable strategy or strategies with one specific feature in total** | - “I tried to remember the words in groups of 3” *(grouping/groups of 3)* - “I repeated the words silently in my mind and then memorized by associating them with something, like apple – eating a delicious apple pie (*rehearsal, association/apple*) |
| **2** | **An identifiable strategy or strategies with two specific features in total** | - “I created images in my mind, like a cat on the sofa and a king in a castle (*association/2 examples*) - “If the word has the same letter such as Groundhog and Goat, I would try and pair them together to make it easier (*grouping/2 examples*)” - “Made links between words, like the cat hopping on the king and the eagle landing on a mountain” (*association/2 examples*) |
| **3** | **An identifiable strategy or strategies with three specific features in total** | - “I tried to make a story in my head, it started with a skunk riding a mule while being chased by an alligator” (*narrative/3 examples*) - “I linked the words with familiar places, like chair – my favourite armchair in our living room, mountain – Mt Snowdon in Wales, castle – the Windsor castle” (*association/ 3 examples*) |
| **4-** | **…and so on** |  |

| **Supplementary Table S2. Descriptive statistics for time taken on the three distractor tasks after each block (seconds).** | | | | | | | | | |
| --- | --- | --- | --- | --- | --- | --- | --- | --- | --- |
|  | **Block** | | **M** | **SD** | | **Min** | | **Max** | |
| **PI 1** | | 33.51 | | | 18.35 | | 13.77 | | 145.48 |
| **PI 2** | | 27.60 | | | 20.53 | | 11.15 | | 192.84 |
| **PI 3** | | 26.63 | | | 16.65 | | 10.01 | | 173.69 |
| **NEW** | | 28.48 | | | 19.47 | | 10.28 | | 167.18 |
| Note. M = mean; SD = standard deviation. PI 1-3 = the proactive interference blocks; NEW = the fourth block with new items that provided release from proactive interference. Participants using more than 100 seconds for the arithmetic distractor task were rare, 2-4 individuals per block. The correlations between distractor task latencies and the rate of correctly recalled words were weak: PI 1 r= -0.08, PI 2 r= -0.04, PI 3 r= -0.003, NEW r= -0.17. | | | | | | | | | |

| **Supplementary Table S3. Average number of correctly recalled words by main strategy type across task blocks.** | | | | |
| --- | --- | --- | --- | --- |
| **M (SD)** | | | | |
| **Strategy** | **PI1** | **PI2** | **PI3** | **NEW** |
| **No strategy** | 6.37 (1.97) | 5.65 (1.74) | 5.23 (1.89) | 6.12 (1.79) |
| **Maintenance** | 7.13 (1.39) | 6.56 (1.97) | 5.74 (2.14) | 7.19 (1.78) |
| **Manipulation** | 7.46 (1.31) | 6.84 (1.69) | 6.58 (1.88) | 7.53 (1.50) |
| Note. PI1-3 = the proactive interference blocks. NEW = the fourth block with new items that provided release from proactive interference. | | | | |

**Supplementary Table S4. Descriptives of intrusion errors by PI block (PI2 and PI3) and main strategy type.**

|  | **PI 2** | **PI 3** |
| --- | --- | --- |
| **NS** | M = 0.31 | M = 0.75 |
|  | SD = 0.70 | SD = 1.00 |
|  | Range = [0; 3] | Range = [0; 4] |
| **MNT** | M = 0.11 | M = 0.55 |
|  | SD = 0.39 | SD = 0.85 |
|  | Range = [0; 2] | Range = [0; 5] |
| **MNP** | M = 0.10 | M = 0.64 |
|  | SD = 0.35 | SD = 0.69 |
|  | Range = [0; 2] | Range = [0; 3] |

**Supplementary Table S5. Sensitivity analyses for strategy type (the PI blocks only)**

| **Predictor** | | **OR (95% HDI)**  **Extra thin priors** |  | **OR (95% HDI)**  **Extra wide priors** |
| --- | --- | --- | --- | --- |
| **Intercept** | | 1.84 [1.47; 2.30] |  | 1.82 [1.44; 2.30] |
| **Block** | | **BF_10_ = 2.131 ⨯ 10^8^** |  | **BF_10_ = 5.527 ⨯ 10^7^** |
|  | PI 2 | 0.78 [0.62; 0.97] |  | 0.78 [0.60; 1.00] |
|  | PI 3 | 0.61 [0.49; 0.77] |  | 0.60 [0.47; 0.78] |
| **Strategy type** | | **BF_10_ = 2.492 ⨯ 10^4^** |  | **BF_10_ = 8.504 ⨯ 10^3^** |
|  | MNT | 1.40 [1.10; 1.79] |  | 1.46 [1.12; 1.91] |
|  | MNP | 1.69 [1.31; 2.18] |  | 1.76 [1.33; 2.32] |
| **Block ⨯ Strategy type** | | **BF_10_ = 0.013** |  | **BF_10_ = 1.156 ⨯ 10^-4^** |
|  | PI 2 ⨯ MNT | 0.98 [0.74; 1.30] |  | 0.96 [0.68; 1.35] |
|  | PI 3 ⨯ MNT | 0.92 [0.68; 1.22] |  | 0.90 [0.64; 1.28] |
|  | PI 2 ⨯ MNP | 0.93 [0.69; 1.25] |  | 0.91 [0.64; 1.30] |
|  | PI 3 ⨯ MNP | 1.06 [0.78; 1.45] |  | 1.06 [0.74; 1.53] |
| Note. Compared to the Cauchy type prior distributions of main results, extra thin priors use scale parameter 0.354 (half); extra wide priors use scale parameter of 1.414 (double). OR = Odds ratio; values over 1 signify better recall, and values under 1 worse recall. Coefficients are from full models. Reference categories are block PI 1 and No strategy use. Bayes factors (BFs) quantify evidence for (BF > 1) or against (BF < 1) the presence of the main effects and the interaction. | | | | |

| **Supplementary Table S6. Sensitivity analyses for level of strategy detail (the PI blocks only)** | | | | |
| --- | --- | --- | --- | --- |
| **Predictor** | | **OR (95% HDI)**  **Extra thin priors** |  | **OR (95% HDI)**  **Extra wide priors** |
| **Intercept** | | 2.56 [2.09; 3.15] |  | 2.63 [2.15; 3.25] |
| **Block** | | **BF_10_ = 4.655 ⨯ 10^4^** |  | **BF_10_ = 8.706 ⨯ 10^3^** |
|  | PI 2 | 0.77 [0.63; 0.94] |  | 0.75 [0.61; 0.92] |
|  | PI 3 | 0.57 [0.46; 0.70] |  | 0.54 [0.44; 0.67] |
| **Level of Detail** | | **BF_10_ = 116.345** |  | **BF_10_ = 52.643** |
|  | Details present | 1.32 [1.04; 1.69] |  | 1.31 [1.00; 1.71] |
| **Block ⨯ Level of Detail** | | **BF_10_ = 0.521** |  | **BF_10_ = 0.066** |
|  | PI 2 ⨯ Details present | 0.98 [0.71; 1.34] |  | 1.00 [0.69; 1.45] |
|  | PI 3 ⨯ Details present | 1.31 [0.93; 1.90] |  | 1.41 [0.95; 2.08] |
| Note. Compared to the Cauchy type prior distributions of main results, extra thin priors use scale parameter 0.354 (half); extra wide priors use scale parameter of 1.414 (double). OR = Odds ratio; values over 1 signify better recall, and values under 1 worse recall. Coefficients are from full models. Reference categories are block PI 1 and No details present. Bayes factors (BFs) quantify evidence for (BF > 1) or against (BF < 1) the presence of the main effects and the interaction. | | | | |

| **Supplementary Table S7. Sensitivity analyses for number of strategies (the PI blocks only)** | | | | |
| --- | --- | --- | --- | --- |
| **Predictor** | | **OR (95% HDI)**  **Extra thin priors** |  | **OR (95% HDI)**  **Extra wide priors** |
| **Intercept** | | 2.67 [2.22; 3.23] |  | 2.69 [2.23; 3.27] |
| **Block** | | **BF_10_ = 1.230 ⨯ 10^5^** |  | **BF_10_ = 2.369 ⨯ 10^4^** |
|  | PI 2 | 0.77 [0.64; 0.93] |  | 0.77 [0.63; 0.93] |
|  | PI 3 | 0.60 [0.50; 0.73] |  | 0.60 [0.49; 0.73] |
| **Number of strategies** | | **BF_10_ = 6.107** |  | **BF_10_ = 2.244** |
|  | Multiple strategies | 1.46 [1.09; 2.00] |  | 1.61 [1.14; 2.30] |
| **Block ⨯ Number of strategies** | | **BF_10_ = 0.252** |  | **BF_10_ = 0.049** |
|  | PI 2 ⨯ Multiple strategies | 0.83 [0.56; 1.19] |  | 0.74 [0.47; 1.15] |
|  | PI 3 ⨯ Multiple strategies | 0.93 [0.62; 1.35] |  | 0.84 [0.53; 1.34] |
| Note. Compared to the Cauchy type prior distributions of main results, extra thin priors use scale parameter 0.354 (half); extra wide priors use scale parameter of 1.414 (double). OR = Odds ratio; values over 1 signify better recall, and values under 1 worse recall. Coefficients are from full models. Reference categories are block PI 1 and Single strategy use. Bayes factors (BFs) quantify evidence for (BF > 1) or against (BF < 1) the presence of the main effects and the interaction. | | | | |
